# Supplementary material for: Splice-Junction-Based Mapping of Alternative Isoforms in the Human Proteome
Source: Cell Rep. Author manuscript; Available in PMC 2020 Jan 15. (PMC6961840; doi:10.1016/j.celrep.2019.11.026)
Supplement: 2 [file NIHMS1546469-supplement-2.zip › DF1/PXD006675/RightVentricle/RightVentricle_49_C1orf43_NTLESTVCTAIYFCEEANHALCNEISK.pdf]

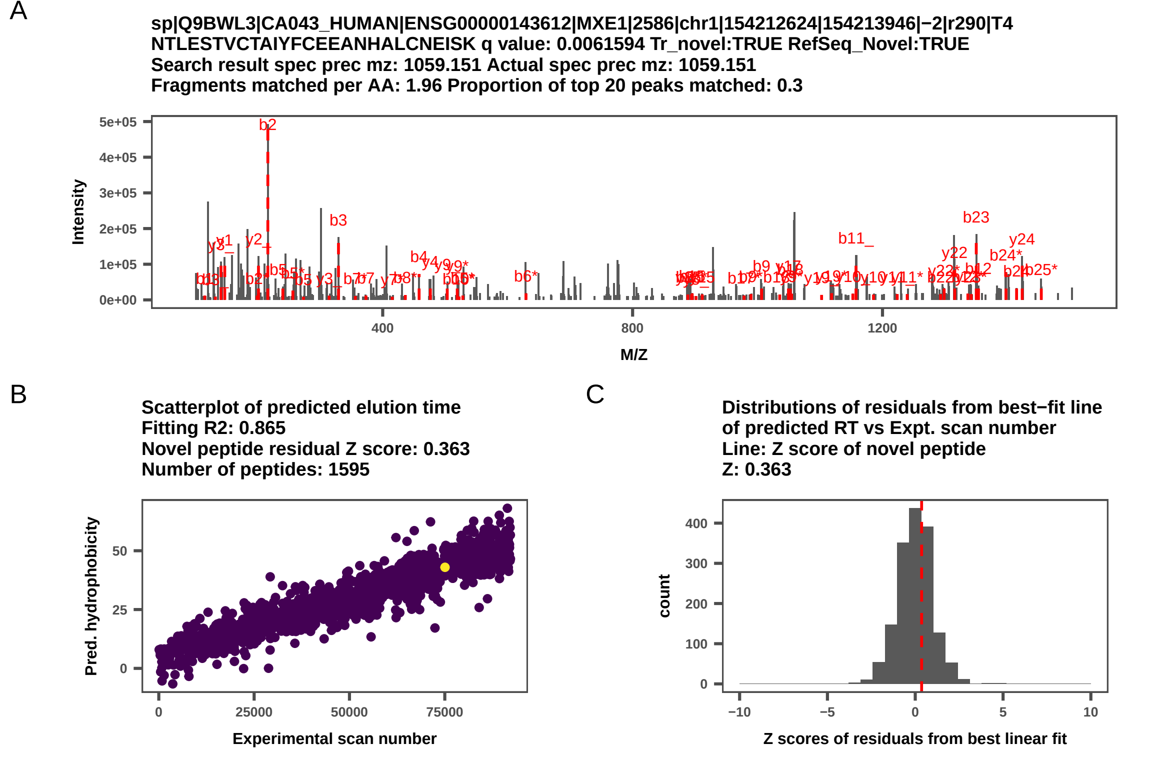
A
sp|Q9BWL3|CA043_HUMAN|ENSG00000143612|MXE1|2586|chr1|154212624|154213946|−2|r290|T4
NTLESTVCTAIYFCEEANHALCNEISK q value: 0.0061594 Tr_novel:TRUE RefSeq_Novel:TRUE
Search result spec prec mz: 1059.151 Actual spec prec mz: 1059.151
Fragments matched per AA: 1.96 Proportion of top 20 peaks matched: 0.3
Intensity
5e+05
4e+05
3e+05
2e+05
1e+05
0e+00
b2
b3
b23
b11_
y24
y22
b24*
y1
y2_
b4
M/Z
400
800
1200
B
Scatterplot of predicted elution time
Fitting R2: 0.865
Novel peptide residual Z score: 0.363
Number of peptides: 1595
Pred. hydrophobicity
50
25
0
0
25000
50000
75000
Experimental scan number
C
Distributions of residuals from best−fit line
of predicted RT vs Expt. scan number
Line: Z score of novel peptide
Z: 0.363
count
400
300
200
100
0
−10
−5
0
5
10
Z scores of residuals from best linear fit
